# Effects of β-mannanase on intestinal health and growth performance of nursery to growing pigs fed diets with DDGS

**DOI:** 10.1093/jas/skaf238

**Published:** 2025-07-29

**Authors:** Hongyu Chen, Yesid Garavito-Duarte, Young Ihn Kim, Shihai Zhang, Sung Woo Kim

**Affiliations:** Department of Animal Science, North Carolina State University, Raleigh, NC 27695; Department of Animal Science, North Carolina State University, Raleigh, NC 27695; Department of Animal Science, North Carolina State University, Raleigh, NC 27695; Department of Animal Science, North Carolina State University, Raleigh, NC 27695; Department of Animal Science, North Carolina State University, Raleigh, NC 27695

**Keywords:** β-mannanase, DDGS, growth performance, intestinal health, pigs

## Abstract

A total of 84 pigs (17.6 ± 2.8 kg initial body weight at 6 wk of age) were used in a 40-d trial to evaluate the effects of dietary supplemental β-mannanase (400 U/kg feed, CTCBIO Inc., Seoul, Korea) on growth performance, digesta viscosity, ileal nutrient digestibility, and intestinal health of pigs during the nursery to grower phase. Pigs were allotted to two treatments (14 pens per treatment, 3 pigs per pen) based on a randomized complete block design with sex as a block. Experimental diets included corn (starter: 50%; grower: 57%), soybean meal (starter: 27%; grower: 20%), and 20% distillers dried grains with solubles with or without 400 U β-mannanase/kg feed. Growth performance (average daily gain, average daily feed intake, and gain-to-feed ratio) was recorded weekly. Plasma was collected on day 35 to quantify tumor necrosis factor-alpha (TNF-α) and malondialdehyde (MDA). On day 35, 0.3% titanium oxide was added as an indigestible marker to the diets for an additional 4-d feeding. On day 40, 16 pigs (1 pig per pen, 8 pens per treatment) were euthanized to collect digesta from jejunum, ileum, and colon (to measure viscosity and pH value) and to collect tissues from duodenum, jejunum, and ileum (for histomorphology, TNF-α, and MDA evaluation). Supplementation of β-mannanase reduced (*P* < 0.05) viscosity of jejunal digesta (2.52 to 1.97 cP, respectively), increased pH of colon digesta (5.99 to 6.33), and tended to reduce (*P* = 0.078) TNF-α concentration (7.94 to 6.46 pg/mg) in jejunal mucosa. Supplementation of β-mannanase decreased (*P* < 0.05) jejunal crypt depth (249 to 212 µm), whereas increasing (*P* < 0.05) ileal villus height (377 to 432 µm) and villus height to crypt depth ratios in both the jejunum (1.58 to 2.10) and ileum (1.65 to 2.02). Supplementation of β-mannanase increased ileal digestibility of neutral detergent fiber (31.3% to 41.1%) and acid detergent fiber (26.8% to 38.7%), whereas improving (*P* < 0.05) G:F ratio during the starter (0.593 to 0.617) and the overall period (0.572 to 0.589). Collectively, dietary β-mannanase (400 U/kg) could improve feed efficiency by decreasing digesta viscosity and increasing nutrient digestibility and could also maintain intestinal health by improving intestinal morphology and reducing inflammatory response.

## Teaser

Supplementing pig diets containing DDGS with β-mannanase reduced digesta viscosity in the small intestine, improved nutrient digestibility, and enhanced intestinal health. These findings highlight the β-mannanase role in optimizing digestion and supporting feed efficiency when feeding pigs during the nursery to grower phase with corn-soybean meal-based diets with DDGS.

## Introduction

Corn and soybean meal are major feedstuffs traditionally used in pig diets due to their nutritional value. However, alternative ingredients such as corn distillers dried grains with solubles (DDGS) have been increasingly utilized, despite their comparatively lower nutritional quality, due to rising costs of conventional feedstuffs (Kim et al., 2019). Corn, soybean meal, and DDGS contain non-starch polysaccharides (NSP), including cellulose, xylan (and arabinoxylan), and β-mannan (and β-galactomannan), as primary components (Pedersen et al., 2014; Tiwari et al., 2018). Soybean meal contains a high level of β-mannan (~2%) (Kiarie et al., 2021), whereas DDGS contains elevated levels of xylan (~20%) and β-mannan (~2%) (Pedersen et al., 2014; Tiwari et al., 2018), which can lead to intestinal challenges in nursery and growing pigs when included at high levels (Chen et al., 2020a; Jang et al., 2024).

The negative impacts of xylan on intestinal health in nursery pigs are well documented (Duarte et al., 2021; Moita et al., 2022; Choi et al., 2024). Similarly, β-mannan has also been associated with detrimental effects on intestinal health in nursery pigs (Baker et al., 2024; Jang et al., 2024). Structurally, β-mannan resembles the outer cell wall of pathogenic bacteria, consisting of mannose units with glucose and galactose side chains (Scheller and Ulvskov, 2010; Tiwari et al., 2018). This structure can be recognized by mannose receptors on the intestinal epithelium, potentially triggering immune activation (Cummings, 2022; Baker et al., 2024).

Pigs consuming feed with increasing levels of selected NSP, including xylan (and arabinoxylan), and β-mannan (and β-galactomannan) show elevated intestinal inflammation and oxidative damage (Lallès et al., 2007; Chen et al., 2020a; Baker et al., 2025). The water-binding capacity of β-mannan enables it to form a gel-like matrix in the intestine, increasing digesta viscosity (Kumar et al., 2012), promoting the proliferation of ammonia-producing bacteria (Agyekum and Nyachoti, 2017; Duarte et al., 2024), slowing passage rate, and reducing the digestibility of key nutrients such as starch, fat, and protein (Gutierrez et al., 2014; Moita and Kim, 2022; Jang et al., 2023), all of which are closely linked to impaired growth performance (Moita et al., 2022; Baker et al., 2024).

To counteract these negative impacts, exogenous β-1,4-mannanases can be included in diets. This enzyme cleaves β-1,4-glycosidic bonds in β-mannans, reducing digesta viscosity in the small intestine, improving nutrient digestibility, and reducing harmful bacteria (Jang et al., 2023; Baker et al., 2024). Additionally, β-mannanase supplementation may exert prebiotic effects by generating oligosaccharides that support beneficial microbiota, enhance short-chain fatty acid (SCFA) production, and improve intestinal health, ultimately enhancing growth performance in poultry and pigs (Agazzi et al., 2020; Baker et al., 2024; Jang et al., 2024). However, the effects of β-mannanase on feed efficiency in pigs remain inconsistent (Carr et al., 2014; Baker et al., 2025). The variability in enzyme efficacy is influenced by multiple factors, including NSP source, diet composition, pig age, feed processing, and enzyme characteristics (Ji and Kim, 2004; Passos et al., 2015; Aderibigbe et al., 2024).

Based on previous findings, this study aimed to test the hypothesis that supplemental β-mannanase in corn-soybean meal-based diets with 20% DDGS will mitigate the negative impacts of NSP, improving nutrient digestibility in pigs during the nursery to grower phase by reducing digesta viscosity, enhancing intestinal immune status by reducing inflammation and oxidative stress, improving ileal nutrient digestibility, and consequently supporting intestinal morphology and improving growth performance.

## Materials and Methods

The experimental protocol was approved by the Institutional Animal Care and Use Committee (REG 10.10.01) of North Carolina State University (Raleigh, NC).

### Animals and experimental design

The experiment was conducted at the North Carolina Swine Evaluation Station (Clayton, NC). A total of 84 pigs at 6 wk of age (42 barrows and 42 gilts) with an initial body weight (BW) of 17.6 ± 2.8 kg were allotted to two dietary treatments. The experimental diets were composed of corn and soybean meal, with 20% DDGS included, either with or without the supplementation of β-mannanase (400 U/kg feed, CTCBIO Inc., Seoul, Korea). Diets were formulated to meet or exceed the nutrient requirements of the NRC (2012), as shown in Table 1. Enzyme activity in the experimental diets was measured post-processing to confirm enzyme stability. One unit of β-mannanase activity was defined as the amount of enzyme that releases 1 μmol of mannose from mannan per minute under 0.2 M sodium phosphate buffer (pH 6) at 50 °C (Jang et al., 2024).

**Table 1.** Composition of experimental diets^1^

Phase β-mannanase, U/kg	Starter		Grower	
	0	400	0	400
Feedstuff, %				
Yellow corn	50.09	50.04	57.48	57.43
DDGS	20.00	20.00	20.00	20.00
SBM	27.20	27.20	20.00	20.00
L-Lys HCl	0.40	0.40	0.30	0.30
DL-Met	0.05	0.05	0.00	0.00
L-Thr	0.06	0.06	0.02	0.02
Sodium chloride	0.22	0.22	0.22	0.22
Vitamin premix^2^	0.03	0.03	0.03	0.03
Mineral premix^3^	0.15	0.15	0.15	0.15
Dicalcium phosphate	0.65	0.65	0.70	0.70
Limestone	1.15	1.15	1.10	1.10
β-mannanase^4^	0.00	0.05	0.00	0.05
Total	100	100	100	100
Calculated composition				
ME, kcal/kg	3,297	3,297	3,302	3,302
SID Lys, %	1.23	1.23	0.98	0.98
SID Met + Cys, %	0.70	0.70	0.58	0.58
SID Trp, %	0.22	0.22	0.18	0.18
SID Thr, %	0.74	0.74	0.60	0.60
Ca, %	0.70	0.70	0.67	0.67
Available P, %	0.34	0.34	0.33	0.33
Analyzed composition				
Dry matter, %	91.33	91.13	91.09	90.97
NDF, %	10.9	11.0	11.7	11.5
ADF, %	5.08	5.36	5.04	5.01
β-mannanase activity^5^, U/kg	0	630	0	790

^1^The experimental period was divided into two phases: starter (1 to 21 d) and grower (22 to 40 d).

^2^The vitamin premix provided the following per kilogram of complete diet: 6,613.8 IU of vitamin A; 992.0 IU of vitamin D_3_; 19.8 IU of vitamin E; 2.64 mg of vitamin K; 0.03 mg of vitamin B_12_; 4.63 mg of riboflavin; 18.52 mg of pantothenic acid; 24.96 mg of niacin; 0.07 mg of biotin.

^3^The trace mineral premix provided the following per kilogram of complete diet: 4.0 mg of Mn as manganese oxide; 165 mg of Fe as ferrous sulfate; 165 mg of Zn as zinc sulfate; 16.5 mg of Cu as copper sulfate; 0.30 mg of I as ethylenediamine dihydroiodide; and 0.30 mg of Se as sodium selenite.

^4^β-mannanase (CTCBIO Inc., Seoul, Korea) at 0.05% replacing corn for treatment diets.

^5^β-mannanase activity: one unit is defined as the amount of enzyme that releases 1 μmol mannose from mannan per minute under 0.2 M sodium phosphate (pH 6) at 50 °C (Jang et al., 2024).

Each treatment had 14 replicate pens (7 barrow pens and 7 gilt pens) with 3 pigs per pen. Pens (4.0 × 1.4 m) with solid concrete floors were equipped with a nipple drinker and a one-hole self-feeder, providing 1.87 m² per pig. Pigs had free access to diets and water. The experimental period was 40 d and was divided into two phases: starter (days 1 to 21) and grower (days 22 to 40). The BW and feed intake were recorded weekly to determine average daily gain (ADG) and average daily feed intake (ADFI). Feed efficiency was calculated as gain-to-feed (G:F) ratio. On day 35, 0.3% titanium oxide was added as an indigestible marker to the diets for an additional 4-d feeding to determine apparent ileal digestibility (AID) of energy and nutrients.

### Sample collection

On day 35, blood samples were collected from the jugular veins using BD sterile vacutainer (BD, Franklin Lakes, NJ) for serum. Blood samples were centrifuged at 3,000 × *g* for 15 min at 4 °C. Serum samples were stored at −80 °C until analysis. Serum samples were used to measure tumor necrosis factor-alpha (TNF-α) and malondialdehyde (MDA).

On day 40, for each treatment, 4 pens from each sex were randomly selected, and 1 pig representing the median BW of each pen (16 pigs in total; 8 pens per treatment) was selected and euthanized via exsanguination following incapacitation with a captive bolt to the head. After euthanasia, the digestive tract was removed to collect digesta samples from the jejunum, ileum, and colon, as well as tissue and mucosa samples from the duodenum, jejunum, and ileum. Jejunal digesta were collected immediately after euthanasia. Samples were then placed in 15-mL tubes on ice for subsequent viscosity analysis. One 50-mL tube of ileal digesta was stored at −20 °C for freeze-drying to calculate the AID of nutrients; another 15-mL ileal digesta tube was placed on ice for pH measurement and viscosity. The colon digesta samples were stored in 50-mL tubes on ice for pH measurement. Mucosal samples from the duodenum, jejunum, and ileum were collected by scraping the intestinal mucosa with a glass slide. The samples were then divided into two 2-mL Eppendorf tubes for TNF-α and MDA measurements, snap-frozen in liquid nitrogen, and stored at −80 °C until analysis. Tissues from the duodenum, jejunum and ileum were rinsed with sterile 0.9% saline solution and stored in 10% formalin buffer at room temperature for intestinal morphology evaluation.

### Viscosity and pH measurement

Viscosity was measured using a viscometer (Brookfield Digital Viscometer, Model DV-II Version 2.0, Brookfield Engineering Laboratories Inc., Stoughton, MA). The tubes containing jejunal digesta were centrifuged at 3,000 × *g* for 5 min, and then 2 mL of the supernatant was centrifuged at 12,500 × *g* for 5 min. Viscometer was set at 25 °C, and 0.5 mL of digesta supernatant was placed in the viscometer. The final result was calculated as the average between viscosity at 45.0/s and 22.5/s shear rates and recorded as centipoise (cP; Duarte et al., 2019; Chen et al., 2020b).

The pH value of jejunal, ileal, and colonic digesta was measured by AB15 Basic pH meter (Thermo Fisher Scientific Inc., Waltham, MA) immediately after sample collection (Choi et al., 2023).

### Inflammatory and oxidative markers

Mucosa samples collected from the jejunum were weighed (1 g) and placed in 1 mL of phosphate-buffered saline on ice. Subsequently, the samples were homogenized using a tissue homogenizer (Tissuemiser; Thermo Fisher Scientific Inc.). The samples underwent centrifugation at 14,000 × *g* for 15 min, following the methodology described by Holanda and Kim (2021). Upon centrifugation, the supernatant was carefully extracted and stored at −80 °C for subsequent analyses. The concentrations of MDA and TNF-α were determined using commercially available kits as per the provided instructions. Optical density (OD) values were measured using a plate reader (Synergy HT, BioTek Instruments, Winooski, VT) and analyzed with corresponding software (Gen5 Data Analysis Software, BioTek Instruments). Concentrations for each analyte were calculated by comparing the resulting OD values against the absorbance of standard curves following the guidelines provided.

Concentrations of MDA in serum and mucosa samples from duodenum, jejunum, and ileum as an index of lipid peroxidation were analyzed using Thiobarbituric Acid Reactive Substance (TBARS) Assay Kit (#STA-330, Cell Biolabs, Inc., San Diego, CA), following the protocol detailed by Moita et al. (2021). Concentrations of MDA in plasma and tissue samples were expressed as μmol/mg protein and μmol/L, respectively. Concentrations of TNF-α in serum and mucosa from duodenum, jejunum, and ileum as a mediator of inflammatory responses were analyzed using Porcine TNF-α Immunoassay ELISA Kit (#PTA00, R&D System Inc., Minneapolis, MN), as detailed in previous work (Cheng et al., 2021). The detection limit range for the TNF-α ELISA was 2.8 to 5.0 pg/mL. Concentrations of TNF-α in plasma and tissue samples were expressed as pg/mL and pg/mg protein, respectively.

### Intestinal morphology

The segments of intestinal tissues were stored in 10% formalin solution and sent to the North Carolina State University histology laboratory for hematoxylin and eosin staining and sectioning according to standard histological technique. The sections were dehydrated and embedded in paraffin. Staining was done using hematoxylin and eosin dyes. Villus height (VH), villus width (VW), and crypt depth (CD) were measured using an Infinity2-2 digital CCD camera attached to an Olympus CX31 microscope (Lumenera Corporation, Ottawa, Canada), and the VH to CD (VH:CD) ratio was subsequently calculated. Lengths of 10 well-oriented intact villi and their associated crypt were measured in each slide, as previously detailed (Jang et al., 2020; Cheng et al., 2021). A single person conducted image collection and analysis of the intestinal morphology samples, and the average result of 10 measurements per pig was reported as a single value per pig.

### Apparent ileal digestibility of energy and nutrients

Diets and freeze-dried ileal digesta were ground and analyzed for dry matter (DM) (Method 934.01, AOAC, 2006). Titanium dioxide concentration in the feed and digesta was measured as previously described by Moita et al. (2021). The gross energy (GE) was obtained using a calorimeter (Model 6200, Parr Instrument Company), detecting energy released during the complete combustion of a sample (1 g). Duplicate samples of diets and ileal digesta were analyzed sequentially for neutral detergent fiber (NDF) and acid detergent fiber (ADF) using the method of Van Soest et al. (1991) in a batch processor (Ankom Technology Corp., Fairport, NY). The AID of DM, GE, NDF, and ADF were calculated using titanium concentration in the diets and digesta as previously described (Chen et al., 2017; Moita et al., 2022). The digestibility was calculated with the following equation:


$$\begin{array}{l} AID,\;\;\;\%\;=\left(1-\frac{TiO2_{diet}\times \;\;\;N_{digesta}}{TiO2_{digesta}\times N_{diet}}\right)\times 100\;\%\;, \end{array}$$

where $TiO2_{diet}$ is the titanium dioxide concentration in the diet, $TiO2_{digesta}$ is the titanium dioxide concentration in the ileal digesta, $N_{diet}$ is the nutrient concentration in the diet, and $N_{digesta}$ is the nutrient concentration in the ileal digesta.

### Statistical analysis

Data were analyzed using the mixed procedure of SAS (SAS Inst. Inc., Cary, NC). Sex was considered a block. The statistical model included β-mannanase supplementation as a fixed effect and sex as a random effect. For growth performance, the pen was the experimental unit, whereas for all other responses, the individual pig (representing each pen) was the experimental unit. A power test was conducted to determine the appropriate number of replications needed for the study to determine statistical significance for an expected mean difference of 7% to 8% at *P* < 0.05, based on previous studies conducted with pigs of a similar genetic background in the same research facility (Baker et al., 2024; Jang et al., 2024). A coefficient of variation of 5% was utilized (Chen et al., 2020b; Kim and Duarte, 2024; Baker et al., 2025). The power of test (1 − beta) at 95% of the power analysis indicated an 80%, the minimum number of replications for each treatment was 14 (Aaron and Hays, 2004). Statistical differences were considered significant with *P* < 0.05 and tendencies with 0.05 ≤ *P* < 0.10.

## Results

### Viscosity and pH of digesta

Pigs fed diets without β-mannanase supplementation had jejunal viscosity of 2.52 cP, whereas pigs fed diets with β-mannanase supplementation showed a lower (*P *< 0.05) jejunal viscosity of 1.97 cP (Table 2). The jejunal pH was 5.69 in pigs fed diets without β-mannanase supplementation and 5.79 in pigs fed diets with β-mannanase supplementation, showing no difference (*P* = 0.610) between treatments. Similarly, the ileal pH was 5.64 in pigs fed diets without β-mannanase supplementation and 5.50 in pigs fed diets with β-mannanase supplementation, with no difference (*P* = 0.387) between treatments. In the colon, the pH in pigs fed diets without β-mannanase supplementation was 5.99, whereas the colon pH in pigs fed diets with β-mannanase supplementation showed an increased (*P *< 0.05) to 6.33.

**Table 2.** Viscosity of jejunal digesta and pH values of jejunal, ileal, and colonic digesta in pigs

Item	β-mannanase, U/kg		SEM	*P-*value
	0	400		
Viscosity, cP^1^				
Distal jejunum	2.52	1.97	0.24	0.041
pH				
Jejunum	5.69	5.79	0.14	0.610
Ileum	5.64	5.50	0.13	0.387
Colon	5.99	6.33	0.08	0.005

^1^cP, centipoise (1 cP = 1/100 dyne s/cm^2^).

### Inflammatory and oxidative markers

Pigs fed diets without β-mannanase supplementation had a serum TNF-α concentration of 88.49 pg/mL, whereas pigs fed diets with β-mannanase supplementation had a serum TNF-α concentration of 77.80 pg/mL (Table 3), showing no difference (*P* = 0.656) between treatments. In the duodenal mucosa, TNF-α levels were 6.70 pg/mg protein in pigs fed diets without β-mannanase supplementation and 7.59 pg/mg protein in pigs fed diets with β-mannanase supplementation, with no difference (*P *= 0.329) between treatments. In the jejunal mucosa, TNF-α concentrations were 7.94 pg/mg protein in pigs fed diets without β-mannanase supplementation, whereas pigs fed diets supplemented with β-mannanase showed a tendency to reduce (*P* = 0.078) TNF-α concentrations to 6.46 pg/mg protein. In the ileal mucosa, TNF-α levels were 5.28 pg/mg protein in pigs fed diets without β-mannanase supplementation and 4.67 pg/mg protein in pigs fed diets with β-mannanase supplementation, with no difference (*P *= 0.262) between treatments.

**Table 3.** Inflammatory and oxidative markers in serum and mucosal sample from the duodenum, jejunum, and ileum in pigs

Item	β-mannanase, U/kg		SEM	*P-*value
	0	400		
TNF-α^1^				
Serum, pg/mL	88.49	77.80	23.29	0.656
Duodenum, pg/mg protein	6.70	7.59	0.88	0.329
Jejunum, pg/mg protein	7.94	6.46	0.74	0.078
Ileum, pg/mg protein	5.28	4.67	0.56	0.262
MDA^2^				
Serum, μmol/L	3.73	3.70	0.39	0.957
Duodenum, μmol/mg protein	0.87	0.75	0.09	0.354
Jejunum, μmol/mg protein	1.31	0.99	0.17	0.101
Ileum, μmol/mg protein	0.79	0.87	0.08	0.459

^1^TNF-α, tumor necrosis factor-alpha.

^2^MDA, malondialdehyde.

Pigs fed diets without β-mannanase supplementation had a serum MDA concentration of 3.73 µmol/L, whereas pigs fed diets with β-mannanase supplementation had a serum MDA concentration of 3.70 µmol/L, indicating no effect (*P *= 0.957) of β-mannanase supplementation on serum lipid oxidation. In the duodenal mucosa, MDA levels were 0.87 µmol/mg protein in pigs fed diets without β-mannanase supplementation and 0.75 µmol/mg protein in pigs fed diets with β-mannanase supplementation, with no difference (*P *= 0.354) between treatments. Similarly, in the jejunal mucosa, MDA concentrations were 1.31 µmol/mg protein in pigs fed diets without β-mannanase supplementation and 0.99 µmol/mg protein in pigs fed diets with β-mannanase supplementation, showing no difference (*P *= 0.101) between treatments. In the ileal mucosa, MDA concentrations were 0.79 µmol/mg protein in pigs fed diets without β-mannanase supplementation and 0.87 µmol/mg protein in pigs fed diets with β-mannanase supplementation, indicating no difference (*P *= 0.459) between treatments.

### Intestinal morphology

In the duodenal tissue, pigs fed diets without β-mannanase supplementation had a VH of 443 µm, whereas pigs fed diets with β-mannanase supplementation had a VH of 454 µm (Table 4), showing no difference (*P *= 0.636) between treatments. Duodenal VW was 132 µm in both treatments, indicating no effect (*P *= 0.869) of β-mannanase supplementation on duodenal VW. The CD was 353 µm in pigs fed diets without β-mannanase supplementation and 327 µm in pigs fed diets with β-mannanase supplementation, with no difference (*P *= 0.207) observed between treatments. The VH:CD ratio was 1.26 in pigs fed diets without β-mannanase supplementation, whereas pigs fed diets supplemented with β-mannanase showed a tendency to increase (*P* = 0.094) the VH:CD ratio in duodenal tissue to 1.40.

**Table 4.** Intestinal morphology of duodenum, jejunum, and ileum tissues in pigs

Item^1^	β-mannanase, U/kg		SEM	*P-*value
	0	400		
Duodenum				
VH, µm	443	454	19	0.636
VW, µm	132	132	5	0.869
CD, µm	353	327	21	0.207
VH:CD ratio	1.26	1.40	0.06	0.094
Jejunum				
VH, µm	379	440	32	0.097
VW, µm	97	101	4	0.485
CD, µm	249	212	17	0.024
VH:CD ratio	1.58	2.10	0.18	0.035
Ileum				
VH, µm	377	432	17	0.026
VW, µm	113	109	4	0.532
CD, µm	233	215	13	0.101
VH:CD ratio	1.65	2.02	0.08	0.013

^*^VH, villus height; VW, villus width; CD, crypt depth; VH:CD, villus height to crypt depth.

In the jejunal tissue, pigs fed diets without β-mannanase supplementation had a VH of 379 µm, whereas pigs fed diets with β-mannanase supplementation showed a tendency to increase (*P* = 0.097) VH in jejunal tissue to 440 µm. Jejunal VW was not different (*P *= 0.485) between treatments, with 97 µm in pigs fed diets without β-mannanase supplementation and 101 µm in pigs fed diets with β-mannanase supplementation. The CD was 249 µm in pigs fed diets without β-mannanase supplementation, whereas pigs fed diets with β-mannanase supplementation showed a reduction (*P* < 0.05) in CD in jejunal tissue to 212 µm. The VH:CD ratio was 1.58 in pigs fed diets without β-mannanase supplementation, whereas pigs fed diets with β-mannanase supplementation showed an increase (*P* < 0.05) in the VH:CD ratio in jejunal tissue to 2.10.

In the ileal tissue, pigs fed diets without β-mannanase supplementation had a VH of 377 µm, whereas pigs fed diets with β-mannanase supplementation showed an increase (*P* < 0.05) in VH to 432 µm. Ileal VW was 113 µm in pigs fed diets without β-mannanase supplementation and 109 µm in pigs fed diets with β-mannanase supplementation, showing no difference (*P *= 0.532) between treatments. The CD was 233 µm in pigs fed diets without β-mannanase supplementation and 215 µm in pigs fed diets with β-mannanase supplementation, with no difference (*P *= 0.101) observed between treatments. The VH:CD ratio was 1.65 in pigs fed diets without β-mannanase supplementation, whereas pigs fed diets with β-mannanase supplementation showed an increase (*P* < 0.05) in the VH:CD ratio in ileal tissue to 2.02.

### Apparent ileal digestibility of energy and nutrients

Pigs fed diets without β-mannanase supplementation had an AID of DM of 75.4%, whereas pigs fed diets with β-mannanase supplementation showed a tendency to increase (*P* = 0.093) the AID of DM to 79.1% (Table 5). The AID of GE was 74.5% in pigs fed diets without β-mannanase supplementation, whereas pigs fed diets with β-mannanase supplementation showed a tendency to increase (*P* = 0.099) the AID of GE to 78.1%. The AID of NDF was 31.3% in pigs fed diets without β-mannanase supplementation, whereas pigs fed diets with β-mannanase supplementation increased (*P* < 0.05) the AID of NDF to 49.1%. Similarly, the AID of ADF was 26.8% in pigs fed diets without β-mannanase supplementation, whereas pigs fed diets with β-mannanase supplementation increased (*P* < 0.05) the AID of ADF to 38.7%.

**Table 5.** Apparent ileal digestibility of energy and nutrients in pigs

Item	β-mannanase, U/kg		SEM	*P-*value
	0	400		
AID, %				
DM	75.4	79.1	1.95	0.093
GE	74.5	78.1	2.00	0.099
NDF	31.3	49.1	4.01	0.001
ADF	26.8	38.7	3.95	0.013

### Growth performance

Pigs fed diets without β-mannanase supplementation had no difference (*P *> 0.05) in growth performance, including BW, ADG, and ADFI, compared to pigs fed diets with β-mannanase supplementation (Table 6). However, during week 1, pigs fed diets without β-mannanase supplementation had a G:F ratio of 0.640, whereas pigs fed diets with β-mannanase supplementation showed a tendency to increase (*P* = 0.093) G:F ratio to 0.697. Similarly, during week 2, the G:F ratio was 0.509 in pigs fed diets without β-mannanase supplementation, whereas pigs fed diets with β-mannanase supplementation showed a tendency to increase (*P* = 0.088) G:F ratio to 0.571. Additionally, G:F ratio during the starter phase was 0.593 in pigs fed diets without β-mannanase supplementation, whereas pigs fed diets with β-mannanase supplementation showed an increase (*P* < 0.05) in G:F ratio to 0.617. The G:F ratio during the overall period was 0.572 in pigs fed diets without β-mannanase supplementation, whereas pigs fed diets with β-mannanase supplementation showed an increase in (*P* < 0.05) G:F ratio to 0.589.

**Table 6.** Growth performance of pigs

Item	β-mannanase, U/kg		SEM	*P-*value
	0	400		
BW, kg				
Initial	17.65	17.62	0.09	0.806
Week 1	21.68	22.05	0.38	0.359
Week 2	25.64	25.96	0.57	0.583
Week 3	30.44	31.68	1.60	0.451
Week 4	37.31	38.19	0.84	0.311
Week 5	43.36	44.22	1.39	0.547
ADG, g				
Week 1	600	642	41	0.328
Week 2	557	605	48	0.330
Week 3	861	817	35	0.228
Week 4	914	930	22	0.626
Week 5	892	862	25	0.406
Starter^1^	672	688	17	0.547
Grower^2^	903	896	16	0.757
Overall	788	792	11	0.802
ADFI, g				
Week 1	943	935	52	0.874
Week 2	1088	1069	52	0.723
Week 3	1399	1376	62	0.721
Week 4	1685	1622	41	0.290
Week 5	2061	2009	51	0.488
Starter^1^	1143	1127	29	0.698
Grower^2^	1873	1815	35	0.266
Overall	1387	1356	24	0.395
G:F ratio				
Week 1	0.640	0.697	0.032	0.093
Week 2	0.509	0.571	0.034	0.088
Week 3	0.625	0.596	0.024	0.258
Week 4	0.544	0.584	0.024	0.252
Week 5	0.434	0.433	0.011	0.981
Starter^1^	0.593	0.617	0.007	0.044
Grower^2^	0.483	0.498	0.008	0.241
Overall	0.572	0.589	0.005	0.037

^1^Starter, days 1 to 21.

^2^Grower, days 22 to 40.

## Discussion

Due to limited endogenous enzyme activity, particularly for degrading complex carbohydrates like NSP, pigs during the nursery-to-grower phase may struggle to efficiently utilize nutrients from high-fiber ingredients such as soybean meal and DDGS (Petry and Patience, 2020; Jang and Kim, 2022). Although corn DDGS is commonly used as a cost-effective feedstuff, its high NSP content, including β-mannans, can impair intestinal health and growth performance in young pigs (Pedersen et al., 2014; Duarte et al., 2019). These soluble NSP increase digesta viscosity in the small intestine, which may inhibit nutrient digestion and absorption by slowing digesta transit, reducing the diffusion rate of substrates, limiting enzyme interaction, and decreasing the available surface area for digestion (Chen et al., 2020a; Duarte et al., 2021; Moita and Kim, 2022). To address this, enzymes like β-mannanase are used in swine nutrition to improve the breakdown of complex carbohydrates in feedstuffs (An et al., 2022). In this study, the inclusion of 400 U/kg of β-mannanase in a corn-soybean meal-based diet supplemented with 20% DDGS reduced the jejunal viscosity by 0.6 cP. The viscosity reduction suggests that a significant portion of the β-mannan in the diet was hydrolyzed by β-mannanase, as observed in previous studies (Baker et al., 2024; Jang et al., 2024). According to Duarte et al. (2021), decreases in jejunal viscosity can facilitate nutrient digestion and absorption in pigs by improving enzyme-substrate interactions and accelerating digesta transit time. Furthermore, Hung et al. (2022) indicated that increased digesta viscosity led to reduced growth performance parameters, impaired intestinal morphology, and reduced amylase activity, suggesting that viscosity is a dominant factor influencing intestinal digestive physiology in pigs. Supplementation with β-mannanase could act by breaking down plant cell walls, releasing encapsulated nutrients, and enhancing the interaction between endogenous enzymes and nutrient substrates (Gomez-Osorio et al., 2021), as was observed by improved ileal digestibility of DM, GE, NDF, and ADF. Additionally, the reduction in jejunal viscosity may increase digesta passage rate, potentially limiting the growth and colonization of pathogenic bacteria by reducing bacterial fermentation (Waldenstedt et al., 2000; Tamargo et al., 2018). The observed increase in digestibility of NDF and ADF with β-mannanase supplementation indicates its role in hydrolyzing β-mannan linkages, producing mannan-oligosaccharides and smaller mannoses, such as mannobiose and mannotriose (Dhawan and Kaur, 2007; Ratnakomala et al., 2022). Although the oligosaccharide composition in the digesta was not measured in this study, previous studies reported that β-mannanase hydrolyzes β-mannan to mannan-oligosaccharides, which have prebiotic effects by modifying the intestinal environment (Mary et al., 2019; Kango et al., 2022). These oligosaccharides have been reported to serve as substrates for beneficial microbiota, including members of the Lachnospiraceae and Ruminococcaceae families, which are associated with increased SCFA production (Fusco et al., 2023; Boston et al., 2024). Such microbial shifts may support intestinal health and potentially inhibit the growth of harmful pathogens such as *Escherichia coli* (Mary et al., 2019; Kumar and Kango, 2021; Firrman et al., 2022). However, future studies measuring microbial populations and SCFA concentrations are needed to confirm these effects in pigs during the nursery to grower phase.

These shifts in microbial fermentation and SCFA production are hypothesized to influence intestinal health and may also affect pH levels along gastrointestinal regions, such as the jejunum, ileum, and colon (Xie et al., 2024), where nutrient absorption processes are critically dependent on maintaining appropriate pH levels. The small intestine is the major site for nutrient absorption; the jejunal pH is critical for effective dissolution, solubilization, and absorption of nutrients (Szabó et al., 2023). The pH ranges of the small intestine (5.7 to 6.8) and the large intestine (5.5 to 6.8) support these processes (Braude et al., 1976). In this study, the pH values fell within the normal range, with no differences except for an increase in colon pH with supplementation of β-mannanase. This finding contrasts with the expectation that fermentation of NSP in the large intestine reduces pH via increased SCFA production (Jha and Berrocoso, 2016). The increase in colon pH could be because β-mannanase hydrolyzed a significant portion of the NSP in the small intestine, thereby reducing the substrate available for fermentation in the colon (Ilhan et al., 2017). Consequently, a reduction in SCFA production in the large intestine may have contributed to the higher colonic pH. However, SCFA concentrations were not measured in this study, limiting the ability to confirm this hypothesis. Future studies incorporating SCFA quantification and microbial profiling in response to β-mannanase supplementation would help clarify the mechanisms underlying pH changes in the colon.

Beta-mannans are recognized by carbohydrate recognition domains of innate immune cells (Ezekowitz, 2003; Tiwari et al., 2020), potentially triggering immune activation. This immune response is marked by increased levels of pro-inflammatory cytokines, such as TNF-α, which can contribute to stress-induced inflammation. Elevated TNF-α levels in the intestinal epithelium disrupt tight junction proteins, leading to mucosal damage and compromised barrier function (Ibuki et al., 2014). Inflammation also promotes favorable conditions for pathogenic microbes by creating an aerobic environment, providing biological sources from sloughed epithelial cells, and ensuring an ideal mucus layer thickness (Lobionda et al., 2019). In this study, β-mannanase supplementation tended to reduce jejunal TNF-α levels, indicating a localized anti-inflammatory effect on the intestine. Because inflammation can drive oxidative damage products through increased production of reactive oxygen species (Ayala et al., 2014), a corresponding antioxidant effect might be expected. However, MDA concentrations suggest that β-mannanase supplementation did not exert an antioxidant effect in the serum or small intestine in this study. This may be due to the relatively low levels of oxidative damage products observed under the experimental conditions, which likely limited the potential for detecting antioxidant effects in the serum and small intestine. These findings are consistent with previous reports on pigs (Ao et al., 2011; Li et al., 2012). Similarly, studies using 20% or 30% DDGS in nursery pig diets showed no effect on lipid peroxidation (Duarte et al., 2019; Chen et al., 2020b), indicating that DDGS inclusion up to 30% did not affect oxidative stress.

The structural integrity of the intestinal epithelium is closely tied to both inflammatory responses and dietary components such as DDGS. Corn distillers dried grains with solubles have been shown to decrease VH and the VH:CD ratio in the ileum, likely due to the interaction of soluble NSP with the mucosa and intestinal microbiota. In the previous studies using pigs with similar genetic backgrounds, pigs fed corn-soybean meal-based diets showed an ileal VH:CD ratio around 1.9, which is considered within the range of healthy intestinal morphology (Chen et al., 2017). This interaction may increase fermentation activity, alter intestinal barrier function, and promote inflammatory responses, which in turn can stimulate crypt-cell proliferation and mucosal protein synthesis (Agyekum et al., 2012; Chen et al., 2020b). In this study, pigs fed a corn-soybean meal-based diet supplemented with 20% DDGS showed a reduced VH:CD ratio in the jejunum and ileum, suggesting a shorter surface area for interaction between nutrients and endogenous enzymes (Chen et al., 2020b). In contrast, supplementation with β-mannanase increased VH in the jejunum and the VH:CD ratio in both the jejunum and ileum, suggesting enhanced intestinal absorptive capacity (Pluske et al., 1997; Jang et al., 2024). These findings indicate that β-mannanase supplementation may support intestinal health by generating mannan-oligosaccharides, which could stimulate enterocyte proliferation through mitogen-activated protein kinase pathways, thereby enhancing epithelial integrity and barrier function (Mavrogeni et al., 2022; Jang et al., 2024). The resulting improvements in intestinal morphology likely contributed to enhanced nutrient absorption and growth performance of pigs during the nursery to grower phase (Pluske et al., 2018), as evidenced by improved nutrient digestibility.

Despite reduced digesta viscosity and improved nutrient ileal digestibility, β-mannanase supplementation did not affect growth performance parameters (BW, ADG, and ADFI). Previous studies have reported inconsistent effects of β-mannanase on growth performance, possibly due to diets being formulated to meet NRC requirements, minimizing the enzyme’s impact (Carr et al., 2014; Upadhaya et al., 2016; Jang et al., 2024). When supplemented with 30% DDGS, neither DDGS nor β-mannanase supplementation improved ADG or G:F ratio during the nursery period (Jones et al., 2010; Kerr et al., 2013). However, improvement in G:F ratio observed in this study suggests better nutrient utilization, which may offer economic advantages through reduced feed cost per unit of gain, despite no observed increase in daily BW gain. Several factors may explain the variable response to β-mannanase in diets, including differences in diet composition, enzyme level and activity, feed processing conditions, and intestinal microbiota composition (Aranda-Aguirre et al., 2021). The physiological status of the pig is also critical; whereas grower–finisher pigs have shown improved ADG with β-mannanase supplementation (Yoon et al., 2010), pigs during the nursery to grower phase may not fully benefit from mannan-oligosaccharides due to their immature digestive systems. Furthermore, the digestive system of young pigs is not fully developed due to their age, making them more sensitive to the NSP challenge (Lindberg, 2014; Jang and Kim, 2022). As a result, the pigs exhibited improved performance when supplemented with exogenous β-mannanase.

In conclusion, β-mannanase supplementation in pigs during the nursery to grower phase, fed a corn-soybean meal-based diet with 20% DDGS effectively reduced jejunal viscosity, apparently demonstrated an anti-inflammatory effect as indicated by reduced jejunal TNF-α levels, and improved intestinal morphology and ileal digestibility of DM, GE, NDF, and ADF. These findings confirm previous studies showing that β-mannanase facilitates the hydrolysis of β-mannans improving nutrient availability. The enhanced intestinal absorptive capacity and digestive function indicate that β-mannanase may support intestinal health in pigs during the nursery to grower phase. These results highlight the potential of β-mannanase to enable greater inclusion of NSP-rich feedstuffs like DDGS in pig diets during the nursery to grower phase without compromising intestinal health or nutrient utilization. Future studies should explore its long-term effects and interactions with intestinal microbiota to optimize its application in pig nutrition during the nursery to grower phase; additionally, incorporating measures of barrier function would further characterize its impact on intestinal integrity.

## Abbreviations

ADF: acid detergent fiber
ADFI: average daily feed intake
ADG: average daily gain
AID: apparent ileal digestibility
BW: body weight
CD: crypt depth
DDGS: corn distillers dried grains with solubles
DM: dry matter
GE: gross energy
G:F: gain to feed
MDA: malondialdehyde
NDF: neutral detergent fiber
NSP: non-starch polysaccharides
SCFA: short-chain fatty acids
TNF-α: tumor necrosis factor-alpha
VH: villus height
VH:CD: villus height to crypt depth
VW: villus width
